# Odorant binding protein 18 increases the pathogen resistance of the imported willow leaf beetle, *Plagiodera versicolora*

**DOI:** 10.3389/fcimb.2024.1360680

**Published:** 2024-02-27

**Authors:** Haoling Rong, Xin He, Yipeng Liu, Mei Liu, Xiaolong Liu, Min Lu

**Affiliations:** State Key Laboratory of Biocatalysis and Enzyme Engineering, School of Life Sciences, Hubei University, Wuhan, China

**Keywords:** odorant binding protein, insect immunity, pathogen infection, pathogen resistance, *Plagiodera versicolora*, *Pseudomonas aeruginosa*

## Abstract

**Background:**

Insect odorant-binding proteins (OBPs) are a class of small molecular weight soluble proteins. In the past few years, OBPs had been found to work as carriers of ligands and play a crucial role in olfaction and various other physiological processes, like immunity. A subset of insect OBPs had been found to be expressed differently and play a function in immunity of fungal infection. However, there are few studies on the role of OBPs in immunity of bacterial infection.

**Methods:**

To identify the immune-related OBPs of *Plagiodera versicolora* after infected by *Pseudomonas aeruginosa*, we determined the mortality of *P. versicolora* to *P. aeruginosa* and selected the time point of 50% mortality of larvae to collect samples for RNA-seq. RNAi technology was used to investigate the function of immune-related OBPs after *P. aeruginosa* infection.

**Results:**

RNA-seq data shows that *PverOBP18* gene significantly up-regulated by 1.8-fold and further RT-qPCR affirmed its expression. Developmental expression profile showed that the expression of *PverOBP18* was highest in the pupae, followed by the female adults, and lower in the 1st-3rd larvae and male adults with lowest in eggs. Tissue expression profiling showed that *PverOBP18* was dominantly expressed in the epidermis. RNAi knockdown of *PverOBP18* significantly reduced the expression of bacterial recognition receptor gene *PGRP* and antibacterial peptide gene *Attacin* and reduced the resistance of *P. versicolora* to *P. aeruginosa* infection.

**Conclusion:**

Our results indicated that *PverOBP18* gene increased the pathogen resistance of *P. versicolora* by cooperating with the immune genes and provided valuable insights into using OBPs as targets to design novel strategies for management of *P. versicolora.*

## 1 Introduction

In the process of long-term evolution, a complex chemosensory system is necessary for insects to find mating partners, locate oviposition sites, forage for foods, and to avoid predators or toxic compounds in their natural environments (Field et al., 2000). Several major steps of the insect chemosensory process are necessary, such as odorant transmission, odorant recognition and insect behavioral guidance. Odorant-binding proteins (OBPs) originally are considered to take the lead in transporting chemical odors from environment to odorant receptors (ORs) of sensory cells, which is the first step of odor recognition (Rihani et al., 2021).

Insect OBP is a small molecular weight odorant carrier protein (15 – 20 kDa), containing approximately 120 to 150 amino acids, belonging to a large family of ligand-binding proteins, and is mainly secreted in sensillum lymph (Pelosi et al., 2018a; Pelosi et al., 2018b). A pheromone binding protein (PBP) found in the antennae of the male *Antheraea polyphemus* is the first member of the OBP family (Vogt and Riddiford, 1981). In the past decades, due to the rapid advancement of genome projects and transcriptome sequencing technologies, a vast number of OBPs have been discovered in various insect species (Venthur and Zhou, 2018; Pelosi et al., 2018a). The quantity of genes encoding OBPs varies significantly across species of insect, ranging from as few as 13 in silkworm *Bombyx mori* to approximately 109 in German cockroach *Blattella germanica* (Sun et al., 2018). However, only a few OBPs have been proved to be exclusively related to olfactory sensing, the functions of most OBPs in insects are still unclear.

Recently, many more OBPs have been reported to be endowed with different functions besides chemo-detection, including insecticide resistance, anti-inflammation and reproduction, as well as immunity (Xiong et al., 2019). For example, several studies indicated that the OBPs in insects were significantly induced by exogenous toxicants and gradually enhanced the resistance of insects to poisons (Gao et al., 2021; Chen et al., 2022). Besides insecticide resistance, the OBPs secreted in mosquito saliva were shown high binding affinity to host biogenic amines and displayed an anti-inflammatory function (Calvo et al., 2009). In addition, some OBPs were identified to be expressed not only in olfactory tissues but also in insect immune tissues such as hemocytes and fat bodies, and participated in innate immunity of insects (Thomas et al., 2016). Certain types of OBPs were found to be induced in insects which may be involved in resistance for fungal infection (Zhang et al., 2017; Zhang et al., 2020; Zheng et al., 2021). For example, in *Locusta migratoria*, the expression of *OBP11* was negatively correlated with larval resistance to *Metarhizium anisopliae* by counteracting the innate immunity of the Toll-pathway (Zhang et al., 2023). These findings suggested that the OBPs played a crucial role in facilitating the immune responses of insects to combat pathogenic fungus.

The *Pseudomonas aeruginosa* is a Gram-negative bacterium and ubiquitous in the environment. It is an opportunistic pathogen and causes a wide range of infectious diseases in diverse host organisms, including insects and humans (Admella and Torrents, 2023; Varponi et al., 2023). *Plagiodera versicolora* (Coleoptera: Chrysomelidae), is a well-known forest pest that poses a threat to plants in the Salicaceae family (Li et al., 2023). In previous study, the chemosensory genes of *P. versicolora* had been identified based on transcriptome of larvae, adult antennas and forelegs, respectively. Collectively, a total of 40 candidate OBPs of *P. versicolora* had been identified (Liu et al., 2021; Wu et al., 2022a; Wu et al., 2022b).

However, there has still been a lack of research on the involvement of OBPs in immunity, particularly concerning bacterial infections. The *P. aeruginosa* was used to infect *P. versicolora*, in order to examine whether OBPs were altering during pathogen infection and carrying out roles in immunity. We first determined the pathogenicity of *P. aeruginosa* to *P. versicolora* and identified the *PverOBP18* gene was immune-induced based on RNA-seq data. RT-qPCR results indicated that *PverOBP18* is highly and specifically expressed in the epidermis. The synergistic toxicity in larvae of *P. aeruginosa* infection and *PverOBP18* knockdown was significantly increased.

## 2 Materials and methods

### 2.1 Insect rearing, pathogen strain and pathogenic infection bioassays

The *P. versicolora* larvae and adults were obtained from Sha Lake Park in Wuhan, China and reared with fresh leaves of willows in laboratory at 28 ± 1°C, under a photoperiod of 12:12 h (light: dark) and 70 ± 5% RH (relative humidity). Strain of *P. aeruginosa* was stored at – 80°C and cultured in Luria-Bertani sterile liquid medium at 37°C for 12 h. OD_600_ was measured to calculate the concentration of *P. aeruginosa* and adjusted it by sterile water to approximately 2.0 for feeding assay. Fresh willow leaves were cut into rectangle and areas were 6 cm^2^. The bacteria solution was added onto the surface of fresh willow leaves for 60 μl and painted repeatedly for several times by plastic coating bar. Fresh willow leaves were air dried with the bacteria concentration was 10 μl/cm^2^ and then fed to the 2nd instar *P. versicolora* larvae that had been deprived of food for 4 h. Synchronized groups of larvae were selected and divided into three groups (each group containing 20 individuals and serving as a biological replicate).

For all pathogenic infection bioassays, the concentrations of the bacteria solutions were consistent with those described above. The experiments were conducted in the artificial climate chamber under controlled conditions as previously described, with willow leaves replaced and survival rate recorded daily.

### 2.2 RNA extraction, cDNA library construction, and Illumina sequencing

Three time points were selected to collected samples, first day, time to reach 10% and 50% mortality. Time to reach 10% and 50% mortality were the time point that the mortality rates of larvae were nearly 10% and 50% (Ma et al., 2021). Considering the dynamics of pathogenicity and lethality in *P. aeruginosa*, the infected group was sampled at day 1, day 2 (time to reach 10% mortality), day 5 (time to reach 50% mortality) after infection and the H_2_O treatment group was sampled at the same time points as controls. Meanwhile, the samples of day 5 were prepared for RNA-seq and the samples of day 1, day 2 and day 5 were prepared for RT-qPCR. Five live larvae in each group were pooled as one biological replication. The whole body of larva was sampled in each treatment.

After collection, all samples were promptly frozen in liquid nitrogen and kept at a temperature of – 80°C for RNA extraction. The total RNA of all collected samples was extracted using Trizol Reagent (Invitrogen, USA) according to protocol. The quality of RNA was checked with a NanoDrop-2000 (Thermo Scientific, USA). The Illumina sequencing of the samples was performed by Majorbio (China). The cDNA library was synthetized with NEBNext ® Ultra mRNA Library Prep Kit for Illumina (NEB, USA) following manufacturer’s instructions and previous study (Liu et al., 2021).

### 2.3 Sequencing assembly, annotation and gene expression analysis

After removing the raw reads containing the adaptor sequences, low-quality reads (Quality score < 20), and repeated reads, the clean reads were obtained. The transcriptome was assembled according to these clean reads by using Trinity 2.8.5 to generate a set of transcripts. These transcripts were annotated according to the following databases: non-redundant (NR) protein database, Swiss-prot, KEGG, KOG/COG, and a search conducted in the National Center for Biotechnology Information (NCBI). A differentially expressed gene (DEG) analysis was performed based on the significance level using the R package, DEGseq2 (p-adjust < 0.05, fold change > 2).

### 2.4 Spatiotemporal gene expression analyses by RT-qPCR

Different stages of *P. versicolora* including eggs, 1st to 3rd instar larvae, pupae, male and female adults were collected respectively. Different tissues of *P. versicolora* including epidermis, foregut, midgut, hindgut and Malpighian tubule were dissected from 3rd instar larvae, respectively (Li et al., 2022). Each sample was studied using three biological replicates. All samples were immediately frozen in liquid nitrogen and stored at – 80°C until used. Following the extraction of RNA, a reverse transcription reaction was carried out using 1 µg of total RNA. The HiScript III RT SuperMix for qPCR (+gDNA wiper) (Vazyme, China) was utilized for cDNA synthesis following the guidelines from the manufacturer. The housekeeping gene *RPS18* was chosen as a reference gene for internal control. Reaction mixtures, reaction protocols and gene expression calculation methods for RT-qPCR have been reported before (Liao et al., 2023).

### 2.5 *In vitro* dsRNA synthesis, RNAi and synergistic toxic bioassays

First, dsRNA template was prepared by PCR using specific primers containing the T7 promoter sequence (**Supplementary Table S1**;10 µM) and purified with a gel extraction kit (Omega, China). Second, dsRNA was synthesized *in vitro* using the T7 RNAi Transcription Kit (Vazyme, China) according to the manufacturer’s instructions and previous study (He et al., 2020). Following the production of dsRNA, two continuous experiments were conducted for synergistic toxic bioassays. First, 1st *P. versicolora* larvae were deprived of food for 4 h before the start of RNAi. Identical amounts (96 ng, in 50 μl H_2_O) of dsRNAs were applied onto fresh willow leaves, covering a consistent area of 6 cm^2^, thus adjusting the concentration of dsRNA to 16 ng/cm^2^. The insects were provided with an adequate amount of fresh leaf coated with dsRNA for RNAi or H_2_O for control. The leaves were replaced daily for three days to ensure that the foliar-applied dsRNA remained stable and target genes were knocked down. Samples were collected on day 3 for RT-qPCR to evaluate the silencing efficiency of RNAi. Second, synchronized groups of 2nd larvae that dsRNA-treated or not were selected, divided into three groups (each group containing 10 individuals and serving as a biological replicate) for pathogenic infection bioassays, and the operations were the same as mentioned above. Samples were collected on day 4 for RT-qPCR to analysis the expression patterns of immune-related genes.

### 2.6 Statistical analysis

The Kaplan-Meier method was utilized to analyze survival curves. The log-rank test was employed to assess the significance of differences between the two groups. The *t*-test was utilized to analyze the data for bacteria induced genes expression and RNAi efficiency by RT-qPCR. The ANOVA was employed to analyze the data for the expression of immunity-related genes by RT-qPCR. A value of *P* < 0.05 meant significantly different. All statistical analyses were performed using SPSS version 22. Figures were drawn with GraphPad Prism 8.

## 3 Results

### 3.1 Survival analysis of *P. versicolora* larvae infected with *P. aeruginosa*

**Figure 1:**
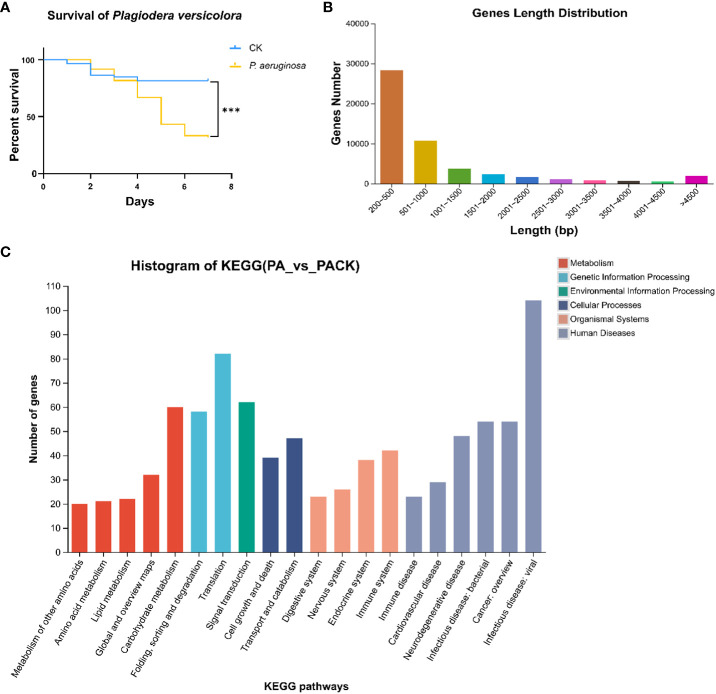
The survival curves and transcriptomic changes of *P. versicolora* larvae infected with *P. aeruginosa* **(A)** Kaplan–Meier survival curves of second-instar *P. versicolora* larvae treated with *P. aeruginosa*. The log-rank test was used to assess the significance of differences between two survival curves. *** P < 0.001. **(B)** Distribution of unigene size in the *P. versicolora* transcriptome assembly **(C)** KEGG classification of differentially expressed unigenes between control (PACK) group and *P. aeruginosa* infected (PA) group.

The survivorship of larvae infected by *P. aeruginosa* was significantly lower than that of control group (**Figure 1A**), demonstrating that *P. versicolora* is very sensitive to this pathogen. After *P. aeruginosa* infection, the mortality rates of *P. versicolora* on day 2 and day 5 were 8.3% and 56.7%, respectively, which were close to 10% and 50%. In comparison, the *P. versicolora* exhibited high survivorship (up to 81.4%) at day 5 in the control group (**Figure 1A**). Therefore, including the first day after *P. aeruginosa* infection, three time points (day 1, day 2, day 5) were selected for further study.

### 3.2 Overview of the sequence assembly

The cDNA library from *P. versicolora* larvae, either infected or not infected with *P. aeruginosa* on day 5, was constructed using the Illumina NovaSeq platform for next-generation sequencing. A total of 59,629,750 clean reads were acquired, with a Q20 percentage of 97.84%. About 51,891 unigenes were discovered, with a combined length of 52453360 bp and an N50 length of 2127 bp (**Table 1**). Statistics showed that 75.2% of the 51,891 unigenes were lower than 1000 bp in length (**Figure 1B**). In total, 20,439 unigenes were matched to entries in the NCBI NR protein database (http://www.ncbi.nlm.nih.gov/protein (accessed on 10 November 2023)) by a BLASTX search.

**Table 1:** Result of the *de novo* transcriptome assembly performed with Trinity.

Type	Unigene	Transcript
Total number	51891	80059
Total base	52453360	97065560
Largest length (bp)	27224	27224
Smallest length (bp)	201	201
Average length (bp)	1010.84	1212.43
N50 length (bp)	2127	2491
E90N50 length (bp)	3887	3290
Fragment mapped percent(%)	72.241	84.346
GC percent (%)	39.95	39.67

### 3.3 Functional classification of KEGG annotation

Overall, the differentially expressed unigenes were categorized into six functional groups based on their KEGG annotation, including environmental information processing (156), human diseases (667), genetic information processing (187), cellular processes (158), organismal systems (347), and metabolism (318). In the functional group of organismal systems, the most differentially expressed genes were found to be associated with the immune system (42) and the endocrine system (38). In the functional group of environmental information processing, the most differentially expressed genes were found to be associated with the signal transduction (62) (**Figure 1C**). The results indicating that some unigenes in these sub-categories might have a connection with chemosensory behavior and immunity in insects.

### 3.4 *PverOBP15* and *PverOBP18* expression were activated during *P. aeruginosa* infection

**Figure 2:**
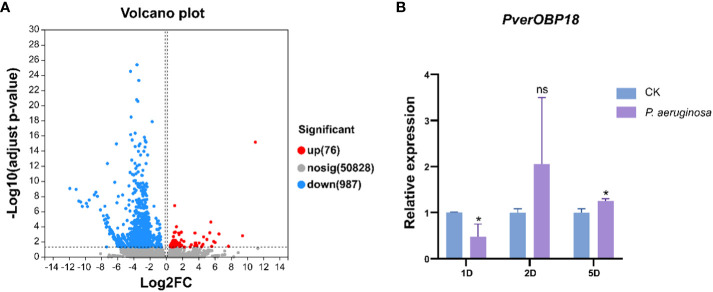
Differential expression analysis between control (PACK) group and *P. aeruginosa* infected (PA) group and the expression profiles of up-regulated gene *PverOBP18*. **(A)** Volcano plot. The log2 FC indicates the mean expression level for each gene. Each dot represents one gene. After *P. aeruginosa* infection, gray dots represent no significant unigenes between PACK and PA, the blue dots represent down-regulated genes and red dots represent up-regulated genes. **(B)** Expression levels of *PverOBP18* gene at three time points, day 1 (1D), day 2 (2D) and day 5 (5D) assessed by RT-qPCR and normalized to the reference gene *RPS18* expression level. Data are means ± SD (n = 3). The letters above the bar indicate the significance of differences as determined by *t*-test. ns, not significant; * P < 0.05.

The transcriptomic studies revealed 11 candidate OBP genes, 2 of which were differentially expressed. *PverOBP15* and *PverOBP18* genes were up-regulated in larvae infected with *P. aeruginosa*, when compared to the control group (**Figure 2A**). In order to investigate whether the transcript level of *PverOBP15* and *PverOBP18* could be triggered by *P. aeruginosa* infection, the expression profiles of these genes were conducted using RT-qPCR at the three previously mentioned time points. The results revealed that the *PverOBP15* gene expression was significantly increased by *P. aeruginosa* infection comparing to control only on day 2, but not day 1 and day 5 (**Supplementary Figure S1**). Unlike *PverOBP15*, the expression of *PverOBP18* was significantly downregulated on day 1. As time went on, the expression of *PverOBP18* was upregulated and significance showed on day 5 (**Figure 2B**).

### 3.5 Expression profiles of *PverOBP15* and *PverOBP18*

**Figure 3:**
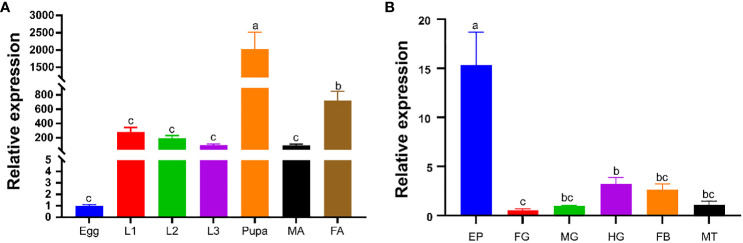
Temporal and spatial expression profiles of *PverOBP18*. **(A)** Expression levels of *PverOBP18* gene at different development stages. L1 - L3, 1st instar larvae – 3rd instar larvae; MA, male adults; FA, female adults. **(B)** Expression levels of *PverOBP18* gene at different tissues of 3rd instar larvae. EP, epidermis; FG, foregut; MG, midgut; HG, hindgut; FB, fat body; MT, Malpighian tubule; Relative expression levels were analyzed using RT-qPCR and normalized to the reference gene *RPS18* expression level. Data are means ± SD (n = 3). The letters above the bar indicate the significance of differences as determined by one-way ANOVA (LSD, P < 0.05).

The relative expression of *PverOBP15* and *PverOBP18* transcripts in different body segments and developmental stages were investigated using RT-qPCR. *PverOBP15* transcript was expressed highest in 3rd larvae and female adults, lower in pupae, and lowest in eggs, 1st larvae, 2nd larvae and male adults (**Supplemetary Figure S2A**). Unlike *PverOBP15*, *PverOBP18* transcript was expressed highest in pupae, followed by female adults, and lower in 1st-3rd larvae and male adults with lowest in eggs (P < 0.05, LSD test) (**Figure 3A**).

In different tissues, *PverOBP15* transcript was expressed highest in the fat body, followed by the foregut, and lowest in the epidermis, midgut, hindgut, and Malpighian tubule (**Supplemetary Figure S2B**). Unlike *PverOBP15*, the expression of *PverOBP18* transcripts was highest in the epidermis, lower in the midgut, hindgut, fat body, and Malpighian tubule, and lowest in foregut (P < 0.05, LSD test) (**Figure 3B**).

### 3.6 *P. versicolora* larvae were sensitive to *P. aeruginosa* infection after knockdown of *PverOBP18*

**Figure 4:**
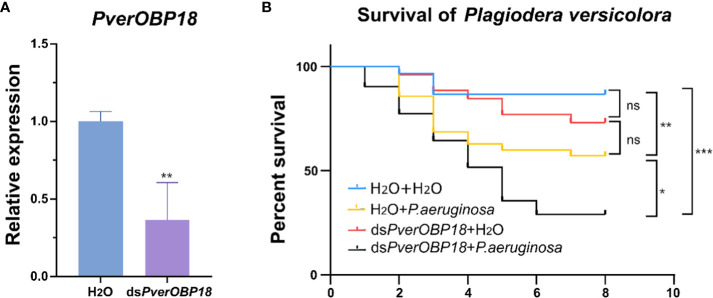
The synergistic effect of dsRNA and the entomopathogenic *P. aeruginosa* on *P. versicolora* larvae. **(A)** Silencing efficiency of *PverOBP18* in *P. versicolora* treated with ds*PverOBP18*. *RPS18* was used as a reference gene to normalize the relative expression of the indicated genes. Data are means ± SD (n = 3). The letters above the bar indicate the significance of differences as determined by *t*-test. ** P < 0.01. **(B)** Comparison of mortality of *P. aeruginosa* infected *P. versicolora* in response to the administration of ds*PverOBP18*. Kaplan–Meier survival curves of second-instar *P. versicolora* larvae. The log-rank test was used to assess the significance of differences between two survival curves. ns, not significant; *** P < 0.001; ** P < 0.01; * P < 0.05.

For the purpose of investigating the role of *PverOBP18* and *PverOBP15* in response to *P. aeruginosa* infection, the dsRNAs for gene knockdown of *PverOBP18* and *PverOBP15* were synthesized *in vitro* and fed to *P. versicolora* larvae. Analyzing the efficiency of RNAi in *P. versicolora*, the results revealed that both *PverOBP15* and *PverOBP18* expression were reduced by nearly 65% after fed dsRNA for 3 days (**Supplementary Figure S3A**; **Figure 4A**). Survival curves showed that the larval mortality of ds*PverOBP18* + ; *P. aeruginosa* treated group was significantly higher than that of the *P. aeruginosa* treated group, while the mortality of ds*PverOBP18* treated group and untreated group were relatively low. Unlike ds*PverOBP18* treatment, no significant difference was found between the ds*PverOBP15* + *P. aeruginosa* treated group and ; *P. aeruginosa* treated group. The results revealed a reduce in the resistance of *P. versicolora* to bacterial infection after the knockdown of *PverOBP18*, but not *PverOBP15* (**Supplementary Figure S3B**; **Figure 4B**).

### 3.7 Validation of immunity-related genes expression patterns by RT-qPCR

**Figure 5:**
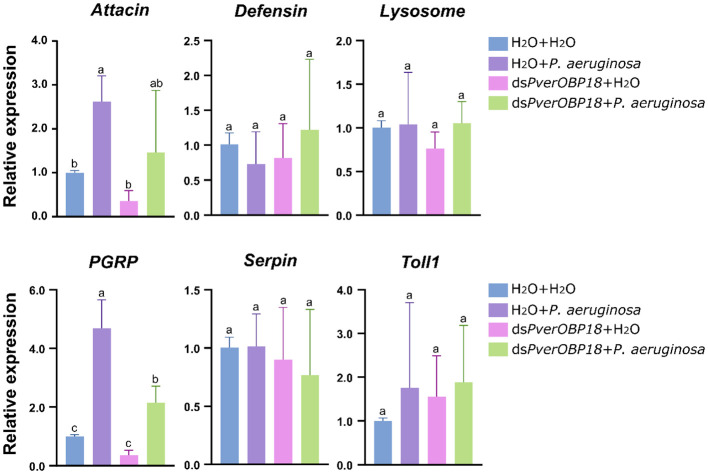
Immunity-related genes analysis of *PverOBP18*-silenced and H_2_O control *P. versicolora* larvae to *P. aeruginosa* infection by RT-qPCR. *PGRP*, peptidoglycan-recognition protein. *RPS18* was used as a reference gene to normalize the relative expression of the indicated genes. Data are means ± SD (n = 3). The letters above the bar indicate the significance of differences as determined by one-way ANOVA (LSD, P < 0.05).

To determine which immunity-related genes were involved in *PverOBP18* mediated resistance to *P. aeruginosa*, genes in immune effectors (*Attacin*, *Defensin*, *Lysosome*), signal modulation (*Serpin*), recognition receptors (*PGRP*) and Toll pathway (*Toll-1*) were selected to analysis the expression patterns by RT-qPCR. The results revealed that *PGRP* gene expression in the *P. aeruginosa* treated group was significantly increased, compared with the ds*PverOBP18* + *P. aeruginosa* treated group, ds*PverOBP18* treated group and untreated group. *Attacin* gene showed a similar expression patten like *PGRP* gene, although there were no notable alterations detected between the *P. aeruginosa* treated group and the ds*PverOBP18* + *P. aeruginosa* treated group. Furthermore, there were no notable alterations detected in the expression of other four immunity-related genes (**Figure 5**).

## 4 Discussion

In this study, *PverOBP18* gene expression was significantly downregulated on day 1 after *P. aeruginosa* infection. As time went on, the expression of *PverOBP18* was upregulated and the significance showed on day 5, suggesting that *PverOBP18* may be involved in *P. aeruginosa* resistance (**Figure 2B**). In nature, because insects are exposed to finite resources and the energetic requirements to trigger immune defense are high, trade-offs often occur between the immune system and other life history traits, such as development, reproduction and physiology (Ardia et al., 2012; Dolezal et al., 2019). For example, in *Drosophila melanogaster*, a series of genes were characterized by a strong decrease in expression at the initial period followed by an increase after the pathogen infection (Schlamp et al., 2021). In *Pieris napi*, several types of immune responses were upregulated during the initial period of *Micrococcus luteus* infection, whereas all other non-immune processes were strongly downregulated (Keehnen et al., 2020). OBPs in insects play important role in binding and transporting chemical odors to odorant receptor neurons (ORNs) through the sensillum lymph during olfaction (Leal, 2013). Therefore, the expression patten of *PverOBP18* induced by *P. aeruginosa* might be a tradeoff between olfactory and immune responses in *P. versicolora*.

In recent years, numerous OBPs in insects had been found to express in both larva and adult (Xue et al., 2016; Liu et al., 2020; Tian et al., 2023). In this study, our results showed that *PverOBP18* gene expression was highest in the pupae, followed by the female adults, and lower in the 1st-3rd larvae and male adults with lowest in eggs. (**Figure 3A**). This indicating that *PverOBP18* may play an important role in various biologic processes during the life stage of *P. versicolora.* Usually, the majority of OBP genes are found to be expressed in chemosensory organs of insectile adults, including the antennae, maxillary palp and proboscis, such as *P. versicolora* (Liu et al., 2021), *Tribolium castaneum* (Dippel et al., 2014) and *Rhynchophorus ferrugineus* (Huang et al., 2023). Previous study had found that *PverOBP18* was mainly expressed in the antennae of adult *P. versicolora* when compared to other tissues, indicating the similar chemoreception function in *P. versicolora* (Liu et al., 2021). However, studies have shown that OBPs are present in various insect tissues and serve functions beyond chemoreception. For example, in *T. castaneum*, *OBPC12* genes was dominantly expressed in the epidermis and displayed high binding affinity to exogenous toxic substances (Gao et al., 2021). Many OBPs in *Lygus lineolaris* demonstrated high levels of expression in the legs and proboscis, indicating their potential roles in chemical detection (Hull et al., 2014). In our study, *PverOBP18* was dominantly expressed in epidermis of 3rd instar larvae, which is an integral part of immune systems and plays a vital role as the first line of defense in insects (**Figure 3B**). These might be the reason why *PverOBP18* increases the pathogen resistance of *P. versicolora* larvae.

In this study, both *PverOBP15* and *PverOBP18* gene expression were activated during *P. aeruginosa* infection (**Figure 2B**; **Supplementary Figure S1**). And the expression of *PverOBP15* and *PverOBP18* in *P. versicolora* were both significantly suppressed after dsRNA treatment (**Figure 4A**; **Supplementary Figure S3A**). However, a significant reduction was showed in *P. versicolora* resistance to *P. aeruginosa* infection just followed by *PverOBP18* RNAi knockdown, but not *PverOBP15* (**Figure 4B**; **Supplementary Figure S3B**). In our comparative analysis, the expression levels of *PverOBP18* were higher than *PverOBP15* in almost all tested tissues and developmental stages (**Figure 3**; **Supplementary Figure S2**). In consideration of the expression level of *PverOBP15* and *PverOBP18*, these might be the reason why the knockdown of *PverOBP15* was useless.

In insects, immune resistance to foreign pathogenic microorganisms relies on the recognition of conserved microbial structures of the microorganisms. For example, peptidoglycan-recognition proteins (*PGRP)* in insects can recognize bacterial surfaces and activate relevant immune pathways to resist bacterial invasion. Recognition of the pathogen leads to downstream reactions, such as antimicrobial peptide synthesis (Eleftherianos et al., 2022). In this study, our results showed that the *PGRP* gene expression level of *P. versicolora* treated with ds*PverOBP18* + *P. aeruginosa* could not reach as high as *P. versicolora* infected by *P. aeruginosa* alone (**Figure 5**). In addition, the *Attacin* gene expression showed a similar result like *PGRP* gene, which is an antimicrobial peptide gene. This means *PverOBP18* gene might relate to *PGRP* gene to influence the recognition of *P. versicolora* to *P. aeruginosa* and further to influence the synthesis of antimicrobial peptide Attacin.

**Figure 6:**
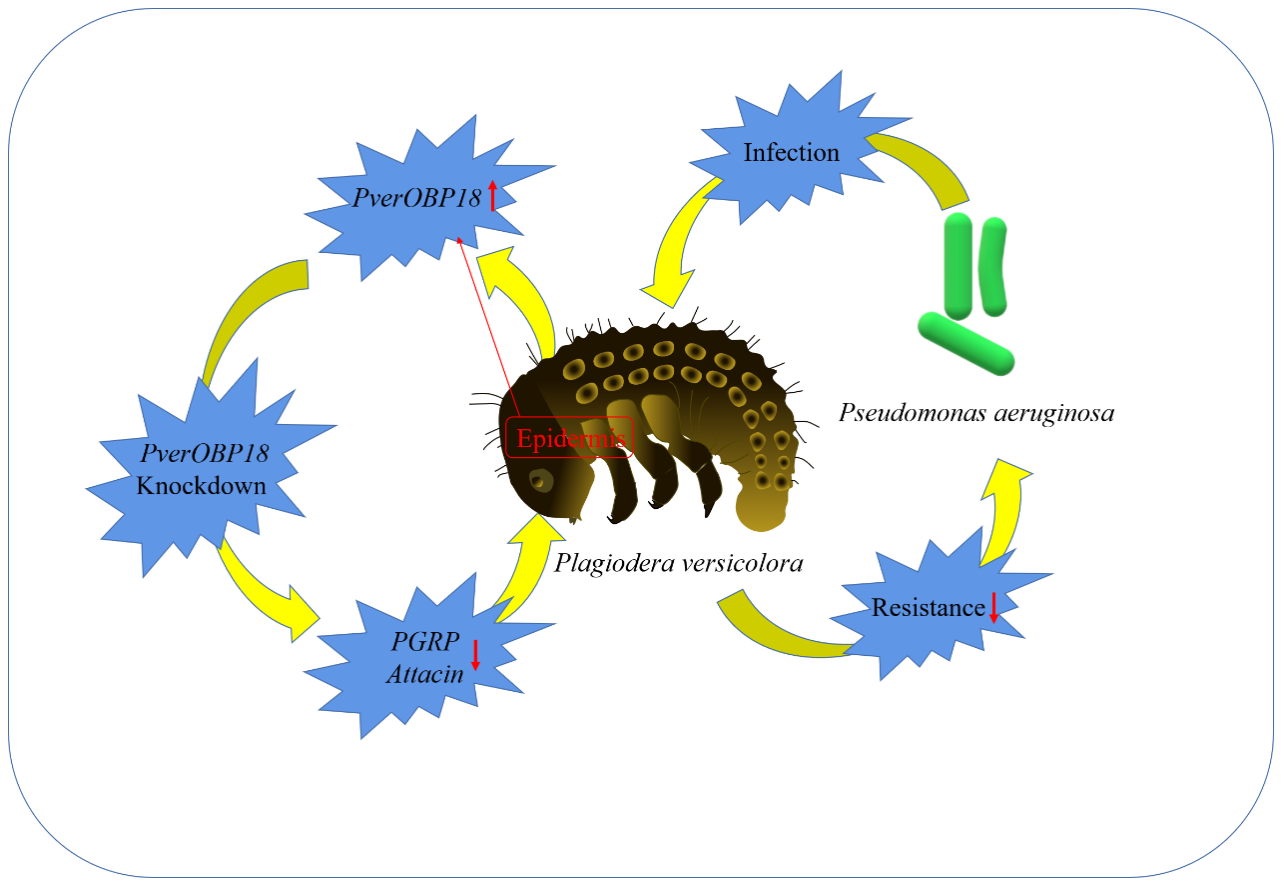
A simple diagram illustrating the mechanism and outcome of how the studied OBP increased the resistance of *P. versicolora* against *P. aeruginosa.*.

In conclusion, the RT-qPCR results showed that the *PverOBP18* gene is highly and specifically expressed in the epidermis. *P. aeruginosa* infection and *PverOBP18* knockdown significantly increased the synergistic mortality in larvae, indicating that the *PverOBP18* gene was clearly triggered. We think these results are broadly relevant and significant, because they (i) show *PverOBP18* is collaborating with immune-related genes to increase *P. versicolora* anti-bacterial resistance, (ii) offer insightful information about using OBPs as targets to develop creative *P. versicolora* management strategies, and (iii) deepen our understanding of OBPs’ functions beyond chemoreception (**Figure 6**).

## Data availability statement

The datasets presented in this study can be found in online repositories. The names of the repository/repositories and accession number(s) can be found below: https://www.ncbi.nlm.nih.gov/biosample/39742880, SAMN39742880 https://www.ncbi.nlm.nih.gov/biosample/39742881, SAMN39742881 https://www.ncbi.nlm.nih.gov/biosample/39742882, SAMN39742882 https://www.ncbi.nlm.nih.gov/biosample/39742883, SAMN39742883 https://www.ncbi.nlm.nih.gov/biosample/39742884, SAMN39742884 https://www.ncbi.nlm.nih.gov/biosample/39742885, SAMN39742885 https://www.ncbi.nlm.nih.gov/biosample/39742886, SAMN39742886 https://www.ncbi.nlm.nih.gov/biosample/39742887, SAMN39742887 https://www.ncbi.nlm.nih.gov/biosample/39742888, SAMN39742888 https://www.ncbi.nlm.nih.gov/biosample/39742889, SAMN39742889 https://www.ncbi.nlm.nih.gov/biosample/39742890, SAMN39742890 https://www.ncbi.nlm.nih.gov/biosample/39742891, SAMN39742891.

## Author contributions

HR: Writing – review & editing, Data curation, Formal analysis, Investigation, Methodology, Software, Writing – original draft. XH: Investigation, Writing – original draft. YL: Resources, Writing – original draft. MeL: Resources, Writing – original draft. XL: Funding acquisition, Methodology, Writing – review & editing. MiL: Funding acquisition, Writing – review & editing, Project administration.
