# Supplementary material for: Odorant binding protein 18 increases the pathogen resistance of the imported willow leaf beetle, Plagiodera versicolora
Source: Front Cell Infect Microbiol. 2024 Feb 27;14:1360680. doi: 10.3389/fcimb.2024.1360680 (PMC10928693; doi:10.3389/fcimb.2024.1360680)
Supplement: Supplementary file 1 [file DataSheet_1.docx]

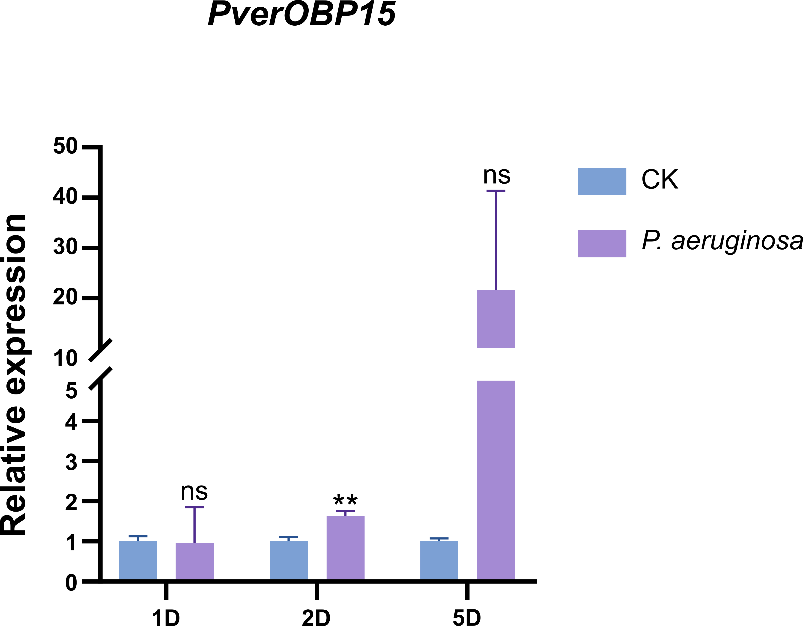


**Figure S1.** The expression profiles of up-regulated gene *PverOBP15*. Expression levels of *PverOBP15* gene at three time points, day 1 (1D), day 2 (2D) and day 5 (5D) assessed by RT-qPCR and normalized to the reference gene *RPS18* expression level. Data are means ± SD (n = 3). The letters above the bar indicate the significance of differences as determined by *t*-test. ns, not significant; ** P < 0.01.


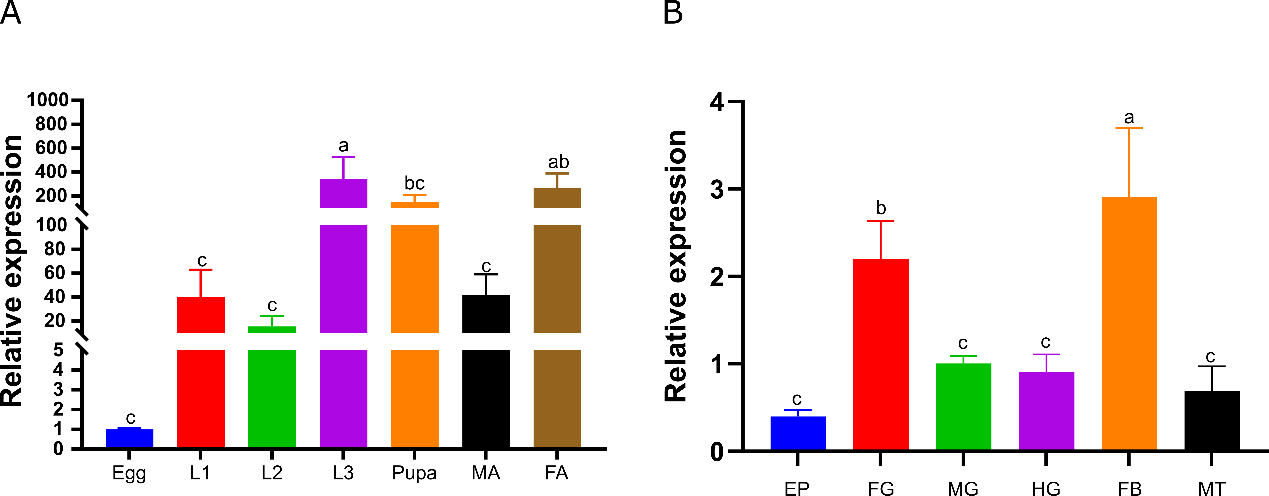


**Figure S2.** Temporal and spatial expression profiles of *PverOBP15*. (A) Expression levels of *PverOBP15* gene at different development stages. L1 - L3, 1st instar larvae – 3rd instar larvae; MA, male adults; FA, female adults. (B) Expression levels of *PverOBP15* gene at different tissues of 3rd instar larvae. EP, epidermis; FG, foregut; MG, midgut; HG, hindgut; FB, fat body; MT, Malpighian tubule; Relative expression levels were analyzed using RT-qPCR and normalized to the reference gene *RPS18* expression level. Data are means ± SD (n = 3). The letters above the bar indicate the significance of differences as determined by one-way ANOVA (LSD, P < 0.05).


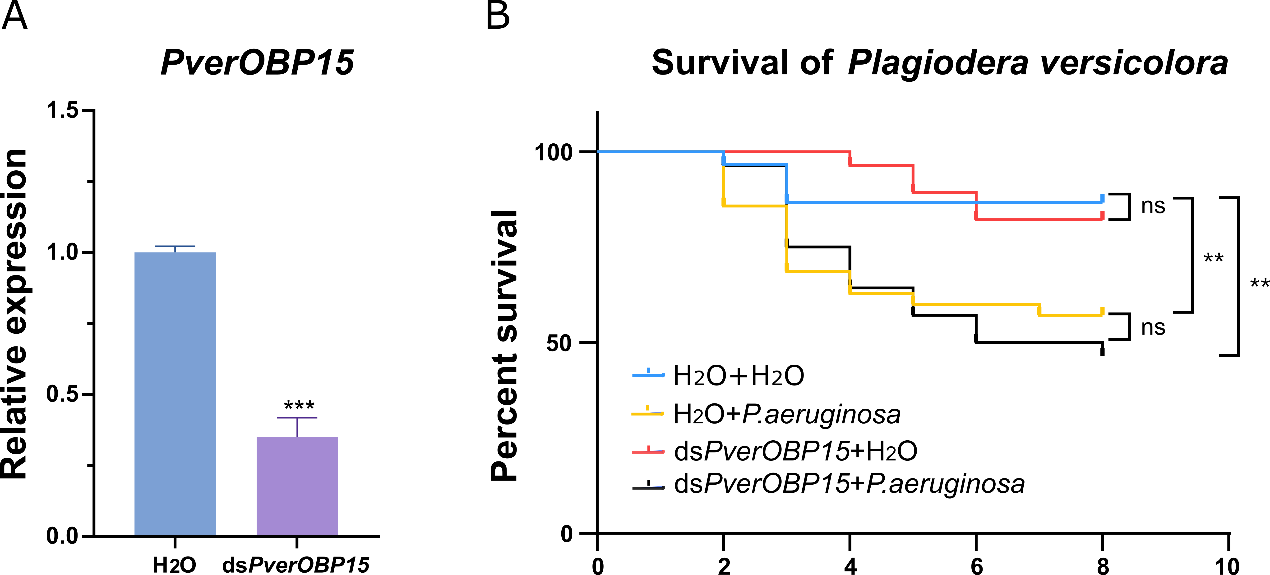


**Figure S3.** The synergistic effect of dsRNA and the entomopathogenic *P. aeruginosa* on *P. versicolora* larvae. (A) Silencing efficiency of *PverOBP15* in *P. versicolora* treated with ds*PverOBP15*. *RPS18* was used as a reference gene to normalize the relative expression of the indicated genes. Data are means ± SD (n = 3). The letters above the bar indicate the significance of differences as determined by *t*-test. *** P < 0.001. (B) Comparison of mortality of *P. aeruginosa* infected *P. versicolora* in response to the administration of ds*PverOBP15*. Kaplan–Meier survival curves of second-instar *P. versicolora* larvae. The log-rank test was used to assess the significance of differences between two survival curves. ns, not significant; ** P < 0.01.

**Table S1.** List of oligonucleotides used in this study. The T7 promoter sequence is indicated in underlines.

| **Oligonucleotide** | **Sequence 5'-3'** | **Description and Use** |
| --- | --- | --- |
| Pv-qRPS18-F | CTTCCTCGTCGGAGCATTCT | forward primer for RT-qPCR analysis of *RPS18* expression (as reference gene) |
| Pv-qRPS18-R | GTTCGCCTTAACTGCCATCAA | reverse primer for RT-qPCR analysis of *RPS18* expression (as reference gene) |
| Pv-qOBP15-F | ACATTTGAGCAGAGCGAGAG | forward primer for RT-qPCR analysis of *OBP15* expression |
| Pv-qOBP15-R | GATAAGGATGCCTTGAGGAAGAT | reverse primer for RT-qPCR analysis of *OBP15* expression |
| Pv-qOBP18-F | TGCACCATTGCATCTTCAAAC | forward primer for RT-qPCR analysis of *OBP18* expression |
| Pv-qOBP18-R | CATACCATCCCTTCCCTTCTTG | reverse primer for RT-qPCR analysis of *OBP18* expression |
| PV-OBP15-T7-F | GGATCCTAATACGACTCACTATAGGCCGACCCAGCAACCAAGATA | forward primer for amplification of the *OBP15* fragment; introducing the T7 promoter sequence; for in vitro dsRNA synthesis |
| PV-OBP15-T7-R | GGATCCTAATACGACTCACTATAGGTCAAGGTGTTCTACTAGGACGAT | reverse primer for amplification of the *OBP15* fragment; introducing the T7 promoter sequence; for in vitro dsRNA synthesis |
| PV-OBP18-T7-F | GGATCCTAATACGACTCACTATAGGCGCAATTGGTCTTGGTAGCC | forward primer for amplification of the *OBP18* fragment; introducing the T7 promoter sequence; for in vitro dsRNA synthesis |
| PV-OBP18-T7-R | GGATCCTAATACGACTCACTATAGGTCAAAAGTGTTCAGAGAAATGACGA | reverse primer for amplification of the *OBP18* fragment; introducing the T7 promoter sequence; for in vitro dsRNA synthesis |
| Pv-qAttacin-F | AAGACACCTGCATCAGGACG | forward primer for RT-qPCR analysis of *Attacin* expression |
| Pv-qAttacin-R | CACTCCTAGTGGTTCTGGCG | reverse primer for RT-qPCR analysis of *Attacin* expression |
| Pv-qDefensin-F | TCCCCGTTCTCTTGGATGAA | forward primer for RT-qPCR analysis of *Defensin* expression |
| Pv-qDefensin-R | CCATGAGACAGGGCAATGGA | reverse primer for RT-qPCR analysis of *Defensin* expression |
| Pv-qPGRP-F | AATTGACTGGACGGTGGTGG | forward primer for RT-qPCR analysis of *PGRP* expression |
| Pv-qPGRP-R | TGGGGTAGAAGTGGGTCTCA | reverse primer for RT-qPCR analysis of *PGRP* expression |
| Pv-qLysosome-F | GAAATGGTGCACTTTCGGCA | forward primer for RT-qPCR analysis of *Lysosome* expression |
| Pv-qLysosome-R | AGCTCTGAAGCCCAACTCTG | reverse primer for RT-qPCR analysis of *Lysosome* expression |
| Pv-qToLL1-F | CACCTCAGTATCAACTTCCT | forward primer for RT-qPCR analysis of *ToLL1* expression |
| Pv-qToLL1-R | CTACGCCGATATGGAGTG | reverse primer for RT-qPCR analysis of *ToLL1* expression |
| Pv-qSerpin-F | TGGCATTCAGAGTAGGATTC | forward primer for RT-qPCR analysis of *Serpin* expression |
| Pv-qSerpin-R | TGACGAGGAAGAAGGACTT | reverse primer for RT-qPCR analysis of *Serpin* expression |
